# Degeneration of Neuromuscular Junction in Age and Dystrophy

**DOI:** 10.3389/fnagi.2014.00099

**Published:** 2014-05-22

**Authors:** Rüdiger Rudolf, Muzamil Majid Khan, Siegfried Labeit, Michael R. Deschenes

**Affiliations:** ^1^Institute of Molecular and Cell Biology, University of Applied Sciences Mannheim, Mannheim, Germany; ^2^Institute of Medical Technology, University of Heidelberg and University of Applied Sciences Mannheim, Mannheim, Germany; ^3^Institute of Toxicology and Genetics, Karlsruhe Institute of Technology, Eggenstein-Leopoldshafen, Germany; ^4^Institute of Integrative Pathophysiology, University Medical Centre Mannheim, Mannheim, Germany; ^5^Department of Kinesiology and Health Sciences, The College of William and Mary, Williamsburg, VA, USA

**Keywords:** neuromuscular junction, dystrophy, aging, sarcopenia, exercise therapy

## Abstract

Functional denervation is a hallmark of aging sarcopenia as well as of muscular dystrophy. It is thought to be a major factor reducing skeletal muscle mass, particularly in the case of sarcopenia. Neuromuscular junctions (NMJs) serve as the interface between the nervous and skeletal muscular systems, and thus they may receive pathophysiological input of both pre- and post-synaptic origin. Consequently, NMJs are good indicators of motor health on a systemic level. Indeed, upon sarcopenia and dystrophy, NMJs morphologically deteriorate and exhibit altered characteristics of primary signaling molecules, such as nicotinic acetylcholine receptor and agrin. Since a remarkable reversibility of these changes can be observed by exercise, there is significant interest in understanding the molecular mechanisms underlying synaptic deterioration upon aging and dystrophy and how synapses are reset by the aforementioned treatments. Here, we review the literature that describes the phenomena observed at the NMJ in sarcopenic and dystrophic muscle as well as to how these alterations can be reversed and to what extent. In a second part, the current information about molecular machineries underlying these processes is reported.

## Introduction

Sarcopenia, a term coined by Rosenberg, literally means “poverty of flesh” and originally referred to aging-related loss of muscle mass (Rosenberg, 1997). The etiology of this loss is most likely multi-factorial. Indeed, the functionality of skeletal muscle is subject to regulation by several different tissues. Primarily, these include muscle, tendons, skeleton, as well as central and peripheral nervous systems (and their supporting cells), but also hormonal glands, blood vessels, and others. Malfunctioning of any one of these components will ultimately affect the other constituents, although with different strength. This complexity of the motor system is certainly an important aspect that leads to the ambiguity in explaining the pathogenic mechanisms of many neuromuscular diseases. That is true even in disorders, in which simple genetic abnormalities are the sole initiating factor, as in the case of hereditary forms of muscular dystrophies. Indeed, although many muscular dystrophies are linked to mutations of different members of a single protein complex, i.e., the dystrophin-associated protein complex (DAPC), insight into the precise links between DAPC and the dystrophic symptoms remains limited. A similar uncertainty characterizes our understanding of the etiology of aging sarcopenia. While there is a general consensus that functional muscle denervation is one of the principal factors leading to sarcopenia, the origin and exact role of the partial silence between nerve and muscle are still debated as outlined below. Since many muscular dystrophies and sarcopenia share defects at the nerve–muscle synapse, i.e., the neuromuscular junction (NMJ), we analyze in this review whether this commonality could possibly reflect similar molecular origins and whether this would allow speculation about diagnostic or therapeutic interventions for these diseases.

## Common Features in Aging and Dystrophy with Respect to the NMJ

Before addressing features that are common or different in muscular dystrophy and sarcopenia, we need to briefly define both terms. *Muscular dystrophies*: these form a group of more than 30 different hereditary or acquired diseases, which are characterized by progressive degeneration of the musculoskeletal system leading in many cases to severe ambulation deficits and reduced lifespan. As excellently reviewed previously (Blake et al., 2002; Davies and Nowak, 2006; Mercuri and Muntoni, 2013), muscular dystrophies can be due to mutations in many different genes, including those encoding sarcomeric/sarcoplasmic proteins like titin or calpain 3, nuclear proteins such as lamin or emerin, and proteins of the sarcoplasmic reticulum like dystrophia myotonica protein kinase. Some forms of muscular dystrophy are also due to defective membrane repair (dysferlinopathy), but the largest part of dystrophies is connected to the DAPC and can be divided into two groups. Diseases originating from mutations in members of the DAPC are often called as dystrophinopathies, while conditions due to aberrant glycosylation of DAPC members (in particular, α-dystroglycan) are termed dystroglycanopathies. Duchenne muscular dystrophy is the most frequent form of dystrophinopathy, Fukuyama congenital muscular dystrophy, and Walker–Warburg syndrome represent typical examples of dystroglycanopathies. Since the DAPC is highly abundant at the NMJ (Pilgram et al., 2009) and might play essential roles in maintaining it (see below), we will limit the discussion in the following text to dystrophinopathies and dystroglycanopathies. *Sarcopenia*: the European Working Group on Sarcopenia in Older People (Cruz-Jentoft et al., 2010), has defined sarcopenia as the loss of muscle mass (atrophy) and muscle strength (dynapenia) as a direct consequence of aging. Given that secondary conditions, like cancer, cirrhosis, or ovariectomy can also lead to (non-sarcopenic) atrophy upon aging, it is not always easy to determine if age or other reasons are the primary cause for muscle loss (Hepple, 2012). This has spurred interest in defining more specific characteristics for the clinical diagnosis of sarcopenia. In the following text, we will examine a few of these criteria (Hepple, 2012) and address to what extent these features are also present in muscular dystrophies.

### Histopathological hallmarks of sarcopenic muscle

Apart from the eponymous loss in muscle mass, sarcopenic muscle is characterized by histopathological traits that can distinguish sarcopenia from other types of muscle atrophy. The first in a list of such parameters is the occurrence of fiber size heterogeneity in muscle from elderly people and analogous mouse/rat models (reviewed in Berger and Doherty, 2010; Hepple, 2012). Notably, while fiber size variability is not found in other types of atrophy like those related to cancer cachexia, it is a major feature of dystrophinopathies (Engel and Ozawa, 2004) and dystroglycanopathies (Taniguchi et al., 2006; Krag et al., 2011; Costa et al., 2013). Second, muscles from elderly exhibit extensive fiber type grouping. This means that a disproportionally large number of neighboring fibers exhibit the same fiber type. This has been found in both humans (Andersen, 2003) and murine models (Kanda and Hashizume, 1989; Rowan et al., 2011). Certainly, fiber type grouping is not very extensive in most muscular dystrophies, but the occurrence of smaller fiber groups was reported for samples from Becker muscular dystrophy (ten Houten and De Visser, 1984; Kaido et al., 1991) and also Duchenne muscular dystrophy (Engel and Ozawa, 2004). Another fiber type-related alteration is the co-expression of multiple myosin heavy chain isoforms, which is again indicative of sarcopenia (Andersen et al., 1999; Patterson et al., 2006; Rowan et al., 2012) and was also observed in Duchenne muscular dystrophy (Marini et al., 1991).

**Figure 1:**
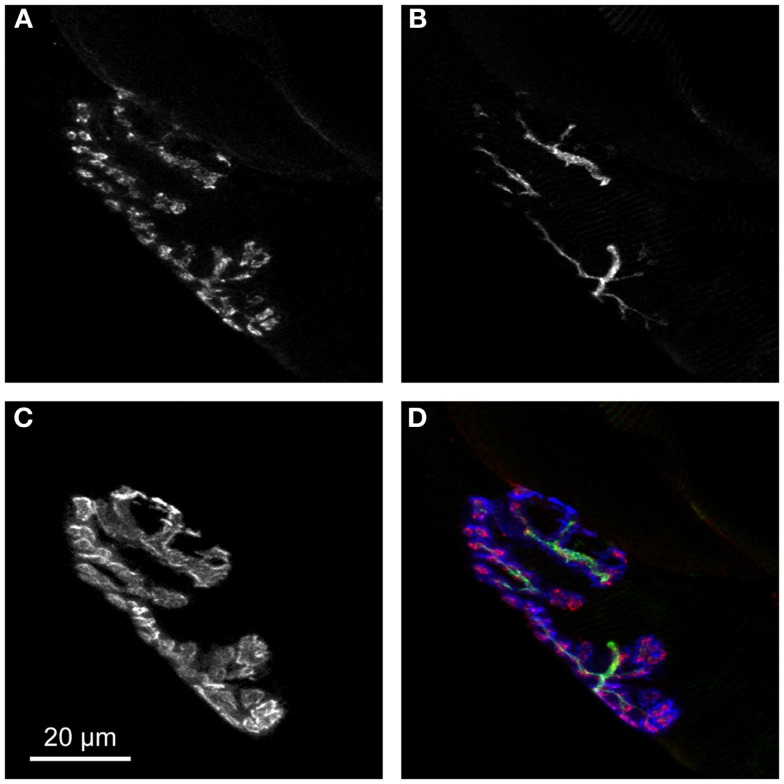
**Pre- and post-synapse exhibit perfect complementarity in young adult NMJs**. **(A,B)** Pre-synaptic terminal as represented by motoneuronal markers for synaptic vesicles [synaptophysin **(A)**] and neuronal cytoskeleton [neurofilament **(B)**]. **(C)** Post-synaptic apparatus contains large amounts of AChRs directly juxtaposed to pre-synaptic ACh filled synaptic vesicles. **(D)** Overlay of synaptophysin (red), neurofilament (green), and AChR stainings (blue) nicely shows the perfect complementarity of pre- and post-synaptic portions of NMJ.

**Figure 2:**
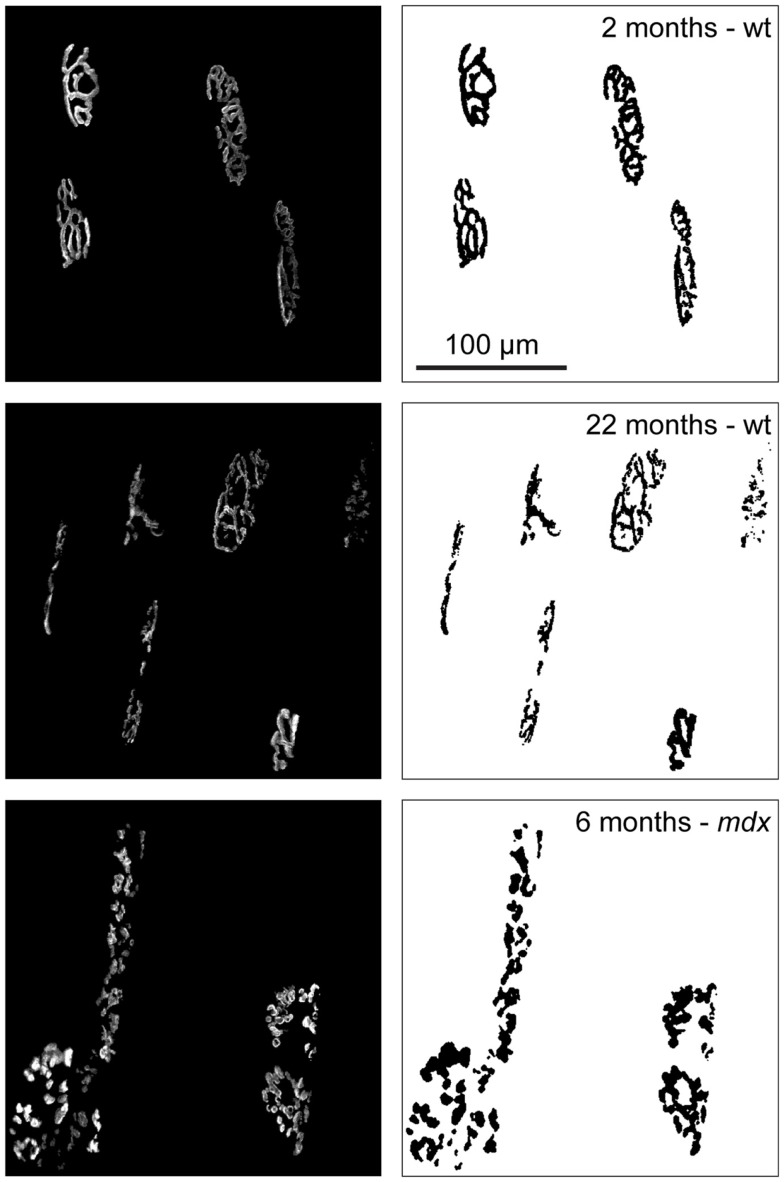
**The phenomenon of NMJ fragmentation during aging and dystrophy**. Tibialis anterior muscles of wildtype (wt) or dystrophic *mdx* mice were injected with fluorescently labeled α-bungarotoxin to mark AChRs at the indicated ages and then imaged using *in vivo* confocal microscopy. Images show maximum intensity projections of several NMJs (left) and binarized pictures (right) to highlight the observation that in young wt AChRs form contiguous, “pretzel-like,” bands while they align in fragmented clusters in aged wt and middle aged dystrophic muscles.

The above-mentioned alterations in fiber size, distribution of fiber type, and co-expression of multiple myosin heavy chain isoforms all suggest the occurrence of reiterating cycles of degeneration/regeneration as well as denervation followed by reinnervation of the affected fibers. Since paucity of neurotransmission is expected to modify the synapses of the involved fibers, it is intriguing that an additional common feature of aging sarcopenia (Valdez et al., 2010; Li et al., 2011) and muscular dystrophies (Lyons and Slater, 1991; Grady et al., 1997; Shiao et al., 2004) is fragmentation of NMJs. But what does fragmentation appear as? In normal mammalian muscle, AChRs densely cluster in winding, band-like arrays on the post-synaptic membrane. Mostly, these bands form a continuous structure also referred to as “pretzel”-like. To achieve maximally efficient neurotransmission, pre- and post-synaptic membranes exhibit the same band pattern (Figure 1). In aged and dystrophic muscle, however, the “pretzel” is fragmented into many individual gutters (Andonian and Fahim, 1987; Lyons and Slater, 1991) (see also Figure 2).

### Origins of synaptic fragmentation might be different

The cause of fragmentation of NMJs in aged and dystrophic muscle is rather unclear, but there are different possible explanations. First, it is conceivable that fragmentation of NMJs is due to degeneration of muscle fiber segments underlying the synapse. Subsequent regeneration would then create a patch-like appearance of the post-synaptic apparatus that could be mimicked by the pre-synaptic terminal through adaptive structural reorganization. This is a principal hypothesis for the occurrence of synaptic fragmentation in muscular dystrophy (Lyons and Slater, 1991) and was also suggested to play a role in the aging process in a longitudinal study in mice, where the same NMJs were followed over extended periods of time (Li et al., 2011). Results showed not a steady, progressive fragmentation, but rather complete fragmentation of individual synapses within short time ranges and mostly in conjunction with the appearance of central nuclei, a sign of regeneration. Feedback from the post- to pre-synaptic regions leading to adaptation of the pre-synaptic terminal to the scattered post-synaptic morphology could be mediated by Lrp4 (Yumoto et al., 2012), the co-receptor of MuSK in agrin-dependent signaling (Kim et al., 2008; Zhang et al., 2008).

Another possible cause of NMJ fragmentation can be traced to the appearance of neuronal lesions or complete motor neuron death leading to muscle fiber denervation followed by reinnervation of the same post-synaptic apparatus by a new neuronal sprout from neighboring neurons. Indeed, in aging sarcopenia, there is wide spread consensus that denervation due to motor neuron apoptosis is a crucial feature. A fast decline in the number of alpha motor neuron cell bodies in the spinal cords of humans beyond the age of 60 was observed (Kawamura et al., 1977; Tomlinson and Irving, 1977; Berger and Doherty, 2010), and was accompanied by a decline in the number of motor units, i.e., the groups of muscle fibers innervated by individual motor neurons (Doherty et al., 1993). Since a massive reduction in the number of muscle fibers in aged muscle was also reported (Lexell, 1993), dying back of motor neurons due to loss of muscle fiber trophic signals could be a possible explanation of sarcopenia. Evidence that aging may induce peripheral nerve degeneration in muscle comes from a recent study in mice, which found signs of marked degeneration of pre- and post-synaptic portions of NMJs although counts of motor neuron somata at the level of the lumbar region did not reveal any difference between mice at 3 and 29 months of age (Chai et al., 2011). Peripheral degeneration of nerves in aged animals was also observed in other reports (Stanmore et al., 1978; Valdez et al., 2010) and investigations from other disease models imply that retrograde motor neuron death is possible (Wong and Martin, 2010). However, in Duchenne muscular dystrophy, motor neuron degeneration among patients has not been detected (Tomlinson et al., 1974) and intramuscular nerves appear normal (Engel and Ozawa, 2004). Given that massive muscle fiber death is occurring in Duchenne patients, it follows that retrograde motor neuron degeneration is not a logical consequence of muscle fiber loss. Finally, smaller rearrangements of molecular events could lead to a steady, slow decay of synaptic regions resulting in fragmented appearance of the synapse although findings in mice do not support this possibility (Li et al., 2011). However, it is unlikely that aging processes in rodents are fully comparable to those in humans, particularly with respect to slowly accumulating ones. In other words, it might well be that small rearrangements that are not visible within the 2–3-year lifespan of mice would in fact be detectable within the more than 80-year lifespan of humans. Before addressing the involvement of different molecular pathways in regulating NMJ morphology and function, we will first address how regular muscle activity affects NMJs. Due to the lack of studies on dystrophic muscle, the ensuing section focuses on the effects of training in healthy, young, and aged subjects and corresponding murine models.

### Role of exercise on muscle performance in aged – focus on NMJ

There is growing epidemiological and experimental evidence suggesting that certain types of physical exercise are effective in offsetting age-related decline in muscle size and strength (reviewed e.g., in Berger and Doherty, 2010). Notably, life-long high-intensity physical activity significantly abates the loss of motor unit numbers (Power et al., 2010). Similar to the way in which chronic exercise training is capable of mitigating the loss of strength and muscle mass associated with aging, the same stimulus of regularly performed exercise appears to influence aging-related adaptations of the NMJ (Andonian and Fahim, 1987). This is evident with respect to both NMJ morphology and function. Moreover, our present understanding suggests that exercise training results in age-specific remodeling of the NMJ. That is, among young adults endurance training elicits an expansion of NMJ dimensions and this enlargement is evident in both the pre- and post-synaptic components of the NMJ. This is not surprising since there is convincing evidence that pre- and post-synaptic relationships of the NMJ are well maintained throughout aging and that this constancy is apparent even when examining the NMJs of muscles displaying different patterns of neuromuscular activity (Deschenes et al., 2013). It should be noted, however, that most of the exercise training studies conducted to date among aged animals have used only moderately aged rodents (i.e., 20–25 months). This is important as it has been reported that during advanced aging (>25 months) rodents display NMJ remodeling that is characterized by reduced dimensions rather than the expansion of synaptic size observed among animals with less advanced aging (Rosenheimer and Smith, 1985). Unclear at this point is how exercise training affects NMJ structure in these more senescent animals and if gender-specific differences occur.

Specific responses of NMJs to exercise training include an increase in total length of nerve terminal branching, a greater number of nerve terminal branches, and a more elaborate pattern of nerve terminal branching, i.e., branching complexity (Fahim, 1997; Deschenes et al., 2011). Accompanying this training-related amplification of nerve terminal branching is a greater total number of pre-synaptic vesicles containing the NMJ’s neurotransmitter acetylcholine (ACh). The greater total nerve terminal branch length in the NMJs of trained muscles is necessary to secure increased numbers of ACh vesicles and total neurotransmitter in light of the fact that the number of vesicles supported by a given length of nerve terminal branching is consistent among young and aged, as well as in muscles with high or low recruitment patterns (Deschenes et al., 2013). It has also been established that the size of individual pre-synaptic vesicles is unaffected by training, again requiring the expression of greater numbers of vesicles if the total amount of stored ACh is to be increased (Fahim, 1997).

As expected in synapses exhibiting tight coupling of pre- and post-synaptic components, endurance training also promotes remodeling with respect to the number and distribution of AChRs at the muscle fiber’s endplate region where post-synaptic depolarization occurs. Specific training-induced endplate modifications are characterized by a higher number of AChRs, which occupy a greater endplate area than in untrained muscle fibers (Deschenes et al., 1993, 2013; Cheng et al., 2013). The enhanced span of the AChR stained area is necessitated by the fact that the density of AChRs anchored within a given area within receptor clusters (i.e., stained area) does not change with training (Deschenes et al., 1993). Thus, to increase the total number of AChRs at the NMJ, the area anchoring these receptors must be expanded. This is precisely what is observed as a result of endurance training.

**Figure 3:**
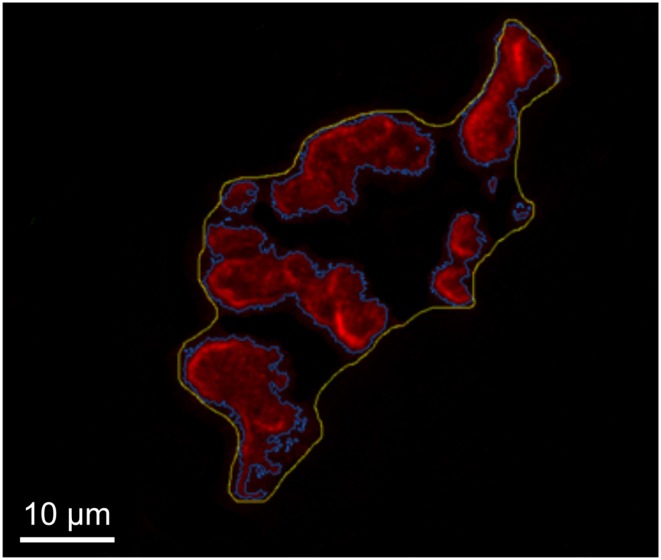
**Determination of post-synaptic ACh receptor cluster dispersion**. Representative image of tracings made to determine total endplate area (yellow) and stained receptors clusters (blue) within that total area. To determine dispersion of AChRs within total endplate the stained area is divided by the total area and multiplied by 100.

Other post-synaptic adaptations induced by training include a greater total perimeter length encompassing the entire endplate region, which includes clusters of receptors as well as interspersing sections of the endplate that do not express receptors. Training has also been found to increase the aggregate perimeter length encompassing only the stained clusters of AChRs without taking into account empty sections between those clusters (Deschenes et al., 2011). Finally, the dispersion of clusters of AChRs within the total endplate area – which includes stained AChR clusters and receptor void sections between those clusters – is sensitive to endurance training, with that stimulus responsible for a more compact, less dispersed distribution of stained receptor clusters (Deschenes et al., 2011). The quantitative technique used to assess the dispersion of AChR clusters within the total endplate area can be seen in Figure 3. As an aside, it is noteworthy that the expanded dimensions detected in run trained NMJs occur despite mild, e.g., 10–15%, atrophy in the size of the muscle fibers on which the NMJs reside (Deschenes et al., 1993, 2011). Clearly, the morphological modifications of NMJs brought about by endurance training cannot simply be attributed to similar remodeling of muscle fiber size.

A basic tenet of the biological sciences is that form and function are inextricably linked. The plasticity of the NMJ is another example of how form and function change hand-in-hand. That is, the endurance training-induced alterations of NMJ structure are associated with significant changes in synaptic transmission. Examples of such electrophysiological adaptations are training-induced elevations in quantal content, or the amount of neurotransmitter released from nerve terminals in response to a single electrical impulse. This is true despite the fact that unstimulated, or random release of ACh, is reduced among trained muscles (Fahim, 1997) suggesting a more secure anchoring of vesicles at pre-synaptic active zones. And during a continuous train of imposed electrical stimuli to the NMJ, there is a slighter degree of depression in post-synaptic response among trained compared to untrained neuromuscular systems (Fahim, 1997). This is viewed as evidence that trained NMJs are more adept not only at recycling the pre-synaptic ACh vesicles releasing neurotransmitter into the synaptic cleft, but also of maintaining sensitivity of the post-synaptic receptors to that ACh which is released.

In contrast to endurance training another form of exercise training, i.e., resistance training, or weight lifting, is known to promote significant muscle fiber hypertrophy (Kraemer et al., 1995). Mimicking the type, intensity, and volume of weight lifting that human athletes routinely perform has proven to be difficult with an animal model. Largely, because of this and because it is not practicable to visually examine the NMJs in human muscle as that synapse is located somewhere in the middle third of the length of the whole muscle and stains must be used to make it visible, much less is known about the effects of resistance training on the NMJ. But in one study using a model of rats climbing a ladder with resistance attached to their tails, it was shown that this mode of exercise brings about NMJ remodeling that is similar in nature to that observed with endurance training, albeit to a smaller extent. More specifically, a 7-week program of resistance training increased pre-synaptic area occupied by ACh containing vesicles, and post-synaptic endplate area stained for AChRs by ~15% – compared to ~30% with endurance training – with the coupling of ACh vesicles and receptors remaining constant (Deschenes et al., 1993, 2000). It appears then, that the stimulus of resistance training, while adequate to result in NMJ remodeling, is not capable of yielding the same degree of synaptic remodeling seen with endurance training.

**Figure 4:**
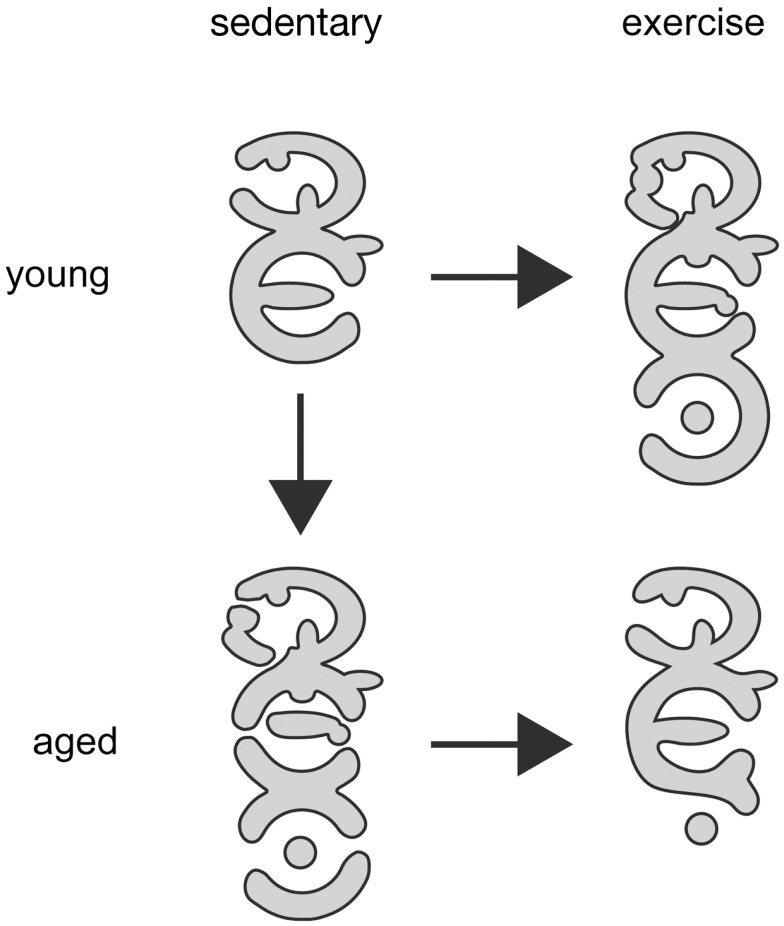
**Schematic representation of exercise-induced changes at post-synaptic membrane of NMJ of young adult and aged rodents**. Both, exercise as well as the aging process come with augmented size of individual NMJs and increased branching complexity of AChR clusters within the NMJ organization. Aged NMJs are also often characterized by enhanced discontinuity of AChR cluster branches. Of note, exercise of aged animals partially reduces branching complexity, NMJ size, and fragmentation and, thus, leads to a morphology of synapses that resembles young adult sedentary NMJs. Relative density of AChR, indicated by gray scales, is always highest at the branch borders and does not significantly change under all conditions. Pre-synaptic branching and synaptic vesicle numbers (not shown) are largely modified in congruency with post-synaptic changes. However, upon aging partial loss of pre-to-post matching does frequently occur.

Although a relatively new area of investigation, a number of studies have reported that aging does, indeed, modify the responsiveness of the NMJ to exercise training. For example, when young adult and aged rats participated in an identical 10-week program of treadmill running, training was shown to result in significant structural remodeling of NMJs (increased nerve terminal total branch length and number of branches, increased post-synaptic area occupied by ACh receptors, and perimeter length surrounding those receptors) in young animals, without such modifications occurring among aged rats (Deschenes et al., 2011). Upon close examination, however, it was apparent that aging alone had resulted in pre- and post-synaptic expansion, so that when aged rats performed endurance training, the effect was to reduce NMJ dimensions back to those observed in untrained young NMJs. Figure 4 depicts the effects of exercise training on post-synaptic structure in young and aged NMJs. A similar age and exercise interaction on NMJ structure has recently been reported (Valdez et al., 2010). In that investigation, it was determined that aged mice displayed larger NMJs with more elaborate, and fragmented, architecture than those examined in young mice. But in aged mice given access to free running wheels, NMJs in exercised muscles were not morphologically distinct from those of young, untrained mice. These studies, along with a more recent report (Cheng et al., 2013), suggest that the morphological remodeling of NMJs associated with aging may be prevented or even reversed through regular participation in endurance type exercise. As aging modulates training-induced structural alterations of the NMJ, exercise-related adaptations in synaptic transmission across the NMJ are similarly affected by aging. Finally, although there is no information regarding the effect of controlled exercise on NMJs in dystrophic muscle, a recent trial documented beneficial impact of moderate bicycle training in a group of Duchenne patients (Jansen et al., 2013).

## Molecular Characteristics and Biomarkers at NMJ

As outlined above, the sequence of events that results in fragmentation of NMJs has yet to be fully revealed. With respect to molecular pathways underlying pre- or post-synaptic processes leading to degeneration of the NMJ there have been recent advancements. Most studies that address the maintenance of the NMJ measure the coherence of the AChR clusters in “pretzel”-shaped arrays. This is mainly for two reasons: first, the AChR is the major ion channel of the post-synaptic apparatus and thus reflects gross morphological alterations of the NMJ with high fidelity. Second, visualization of its distribution, also *in vivo*, is greatly facilitated by the use of the snake venom, α-bungarotoxin, which does not permeate the cell membrane, but binds with extremely high specificity and affinity to AChRs and can be labeled with a variety of dyes, radioactive tracers, etc. To better understand possible scenarios that could affect NMJ morphology in general and AChRs in particular, it is useful to mention the principal steps of AChR lifecycle.

### Biogenesis and clustering of AChRs

**Figure 5:**
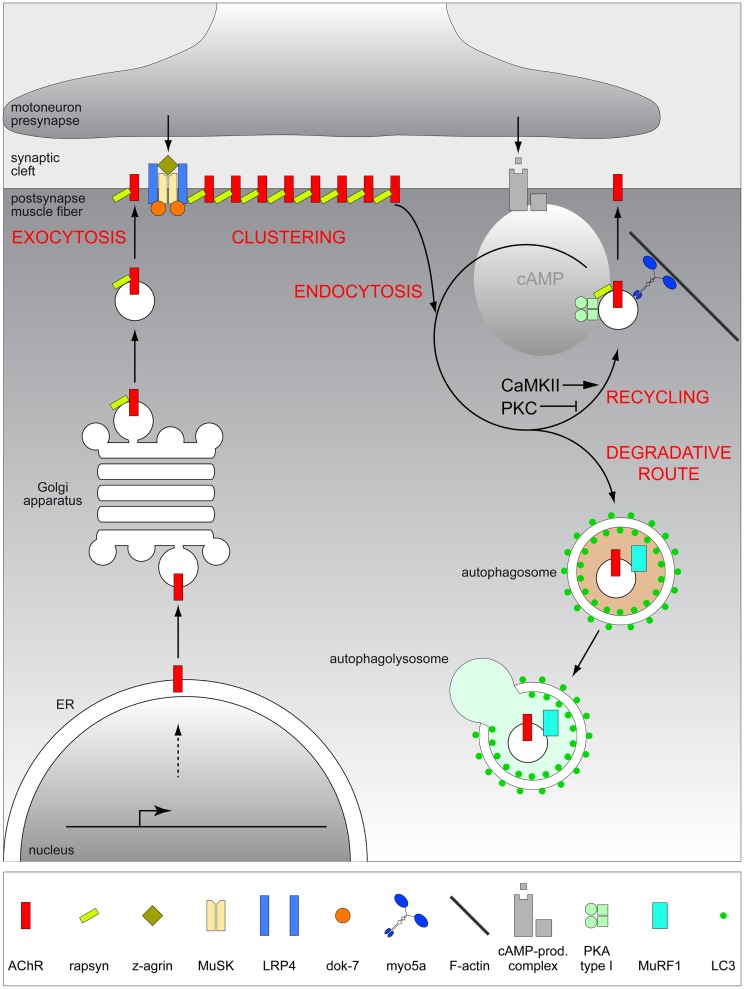
**Scheme depicting the putative life cycle of AChR at the vertebrate NMJ**. Upon assembly in the ER and glycosylation in the Golgi apparatus, AChR is delivered by exocytosis to the post-synaptic membrane where it is clustered by means of agrin/MuSK/Lrp4/rapsyn complex, to fulfill its major function, i.e., mediating neuromuscular transmission. Subsequently, AChR is endocytosed and can then be recycled using a cooperative function of myosin Va, PKA type I, and rapsyn, or degraded, presumably via autophagic decay in a MuRF1-dependent manner. Decision-making between recycling or degradation appears to be subject to manifold signals including CaMKII and PKC.

The AChR is a pentameric ligand-gated cation channel of the cys-loop family of ion channels with a wide expression in the central nervous system and skeletal muscle. In the latter, AChR subunit composition depends on the developmental state of the tissue. While embryonic and regenerating muscles express AChRs with a subunit composition of αγαδβ, adult intact muscle expresses receptors with αεαδβ composition (Witzemann et al., 1987, 1989; Gu and Hall, 1988). By substituting the ε subunit for the γ subunit, the gating properties of the channel change from displaying long open times with slow conductance, to brief open times with high ion conductance rates (Mishina et al., 1986; Schwarz et al., 2000). The γ to ε switch was found to be essential, because mice lacking the adult-type AChRε subunit showed impaired neurotransmission, progressive muscle weakness, and died about 40–60 days after birth (Witzemann et al., 1996). The AChR life cycle starts with biogenesis and assembly of its subunits within the endoplasmic reticulum and then proceeds in the Golgi apparatus, where glycosylation occurs, before secretory vesicles transport the AChR to the post-synaptic membrane (Figure 5). Already during this transport, the 43 kDa receptor associated protein of the synapse (rapsyn) escorts the AChRs (Marchand et al., 2000, 2002) in a 1:1 fashion. At the membrane, AChRs are then clustered and maintained by the help of rapsyn that links AChRs to the underlying actin cytoskeleton via the DAPC (Gautam et al., 1995). This process is mainly mediated by the agrin/MuSK/Lrp4 signaling pathway (Figure 5) that is regulated by the presence of an active motor neuron pre-synaptic terminal. As recently reviewed (Punga and Ruegg, 2012), the large proteoglycan, neural agrin, activates the MuSK–Lrp4 complex to inhibit activity-dependent AChR cluster disassembly that occurs outside the reach of neuronal agrin. This involves the activation of effector molecules, primarily Dok-7 and rapsyn. Thus, the agrin/MuSK/Lrp4 pathway appears to perfectly explain the observed precise fit of pre- to post-synaptic portions of healthy NMJs by release of a neuronal signal and the subsequent response of muscle tissue. However, at least two factors complicate this concept. First, the agrin/MuSK/Lrp4 pathway is not the only regulator of AChR clustering. Indeed, a wealth of other signaling molecules also affects the presence of AChRs at the membrane, as reviewed previously (Wu et al., 2010). Second, it is increasingly appreciated that NMJ development and maintenance are not controlled in a simple unidirectional manner from nerve to muscle. For example, while agrin is dispensable for AChR cluster formation during development, cluster maintenance in the adult needs agrin to prohibit activity-induced cluster dispersal (see Kummer et al., 2006 for review). Furthermore, muscle-derived Lrp4 appears to also act in a retrograde manner, since it is necessary for early steps of pre-synaptic development *in vitro* and *in vivo* (Yumoto et al., 2012). In the context of aging research, it would be very interesting to know, whether Lrp4 also signals from muscle to nerve in adult tissue to maintain the nerve–muscle connectivity intact in a retrograde manner.

### Life or death after leaving the cluster

Although AChRs in intact and innervated muscle exhibit a high metabolic stability and are, thus, slowly turned over with a half-life of about 13 days, they are at some point removed from the post-synaptic region by endocytosis. Subsequent fates include storage in an intracellular compartment, recycling to the post-synaptic membrane in an activity-dependent manner, and degradation (Figure 5). Given that about 25% of all surface receptors are normally recycled within 4 days (Bruneau et al., 2005), positive or negative tuning of the decision between recycling and deterioration would also affect AChR density at the NMJ, and if recycling is lacking spatial precision, this could potentially lead to fragmentation of synapses due to receptor delivery at wrong sites. A couple of reports indicate that second messenger-triggered serine/threonine phosphorylation events are major determinants controlling the decision-making between recycling and degradation. Observations from different laboratories have revealed the positive and negative impacts of protein kinases A (PKA) and C (PKC), respectively, on AChR lifetime and recycling (Nelson et al., 2003; Röder et al., 2010; Martinez-Pena y Valenzuela et al., 2013). Furthermore, Ca^2+^/calmodulin-dependent kinase II (CaMKII) also appears to trigger AChR recycling in an electrical stimulation and Ca^2+^-dependent manner (Martinez-Pena y Valenzuela et al., 2010) (Figure 5).

The target molecules for these phosphorylation events are still unclear, but one obvious candidate is the AChR itself, which can be phosphorylated at different subunits by PKA and PKC (Miles et al., 1987; Huganir and Miles, 1989; Nimnual et al., 1998). A potential target for CaMKII at the NMJ could be myosin Va, which is crucial to recruit AChR-containing recycling carriers to the post-synaptic membrane (Röder et al., 2008; Yampolsky et al., 2010). Myosin V molecules are CaMKII-regulated motor proteins (Costa et al., 1999) and are also important for activity- and Ca^2+^-dependent recycling of AMPA receptors at central synapses (Correia et al., 2008; Wang et al., 2008). Furthermore, myosin V is dependent on actin filaments to escort vesicles to their destination (Reck-Peterson et al., 2000; Walker et al., 2000). This might partially reflect the aberrant recycling of AChRs upon impairment of components of the DAPC (Martinez-Pena y Valenzuela et al., 2011; Schmidt et al., 2011), which is a major organizer of the actin cytoskeleton in skeletal muscle and the NMJ (Pilgram et al., 2009). Another general point that characterizes the organization of AChR recycling is the compartmentalization of signaling by virtue of anchoring molecules. While the cAMP necessary to recycle AChRs in a PKA-dependent manner appears to be located in certain microdomains (Röder et al., 2010), rapsyn was found to anchor PKA in close vicinity to the vesicles that harbor recycling AChRs and myosin Va (Röder et al., 2010; Choi et al., 2012). Similarly, absence of the CaMKII-anchoring protein αkap (which itself is a non-functional CaMKII), that is known to target CaMKII to different subcellular sites (Bayer et al., 1998; O’Leary et al., 2006; Singh et al., 2009), reduced AChR stability in myotubes (Mouslim et al., 2012). The presence and absence of αkap was claimed to be associated with decreased and increased ubiquitination, of AChRs (Mouslim et al., 2012), respectively, and regulation of this process might be directly linked to degradation of AChRs. While in this study, using myotubes and cell cultures, total AChR levels could be modulated using proteasome inhibitors (Mouslim et al., 2012), other reports show a role of lysosomal degradation of surface-exposed AChRs (Libby et al., 1980; Engel and Fumagalli, 1982; Clementi et al., 1983; Hyman and Froehner, 1983; Valkova et al., 2011). A likely explanation for the discrepancy regarding the degradation path of AChRs is the time-point at which they are to be eliminated. Whereas proteasome appears to be crucial for degradation of unassembled AChR subunits at the level of ER (Christianson and Green, 2004), lysosomal degradation should affect receptors after their endocytosis from the plasma membrane.

A recent study has looked at this latter degradation pathway in more detail and reported the involvement of autophagy that leads to loss of AChRs from the NMJ (Khan et al., 2014) (Figure 5) after ingestion of endosomal structures into autophagosomes that subsequently fuse with lysosomes to terminally digest the entire protein content as reviewed elsewhere (Shaid et al., 2013). AChR-containing endocytosed carriers were accompanied by the autophagy marker, LC3 and this was dependent on the presence of the LC3-activating enzyme, Atg7 (Khan et al., 2014). Notably, a strong increase in the amount of autophagic AChR-containing vesicles upon denervation was observed and this was completely blunted in the absence of the E3 ubiquitin ligase, MuRF1 (Khan et al., 2014). MuRF1 is also known as one of the central players in muscle atrophy (Bodine et al., 2001; Centner et al., 2001) and termed as atrogene (Lecker et al., 2004). This suggested a role of ubiquitination in sorting AChRs to autophagic decay, an assumption which was corroborated by the presence of the adaptor protein, p62/SQSTM1 in AChR-containing carriers (Khan et al., 2014). p62/SQSTM1 harbors both, ubiquitin binding site and LC3 interacting regions to bridge ubiquitinated target molecules to the autophagosomal membranes (Pankiv et al., 2007). So far, it is unclear, if ubiquitinated AChR serves as a direct target for selective autophagy or whether other molecules in complex with AChR do so.

### Pathways altered in aging and dystrophy

The above discussion has mentioned a few molecular pathways that could potentially play a role in the deterioration of the NMJ. In the following text, actually observed alterations of these pathways in aging and dystrophy will be discussed. A recent genomic and proteomic profiling of aging rats revealed that a group of NMJ-related genes, including different subunits of AChR, MuSK, and Lrp4, are significantly up-regulated with age and weight loss (Ibebunjo et al., 2013). This has two implications. First, it corroborates an important involvement of functional denervation in the sarcopenia process, since synaptic genes are known targets upon denervation (Bodine et al., 2001; Furlow et al., 2013). Second, the MuSK pathway is apparently involved in the aging process of skeletal muscle. This is further substantiated by recent studies. First, tamoxifen-inducible conditional knock-out mice lacking agrin in a subset of motor neurons were recently created and analyzed (Samuel et al., 2012). Upon tamoxifen treatment of these mice, it took 2–3 months for agrin to detectably diminish in the affected neurons. Concurrently, NMJs displayed mild to severe morphological alterations. Notably, pre-synaptic decay seemed to follow deterioration of the post-synaptic apparatus, suggesting the involvement of retrograde signaling from muscle to nerve (Samuel et al., 2012). Five to six months after the administration of tamoxifen, there was marked withdrawal of motor axons and motor unit sizes were decreased (Samuel et al., 2012). Another important contribution was made by the finding that proteolytic cleavage of agrin induces early onset sarcopenia in young adult mice (Bütikofer et al., 2011). Agrin is cleaved at the NMJ by the protease, neuronal neurotrypsin, and this leads to the formation of 90 and 22 kDa N- and C-terminal fragments of agrin, respectively (Reif et al., 2007; Stephan et al., 2008). Notably, 4 months old transgenic mice overexpressing neurotrypsin in motor neurons displayed many facets of sarcopenia including reduced fiber number, fiber caliber heterogeneity, fiber type grouping, increased amount of type I fibers, and severely fragmented NMJs (Bütikofer et al., 2011). These findings suggest that destabilization of NMJ alone can be sufficient for leading to a pronounced sarcopenic phenotype and were substantiated by a recent study, where injection of a neurotrypsin-resistant agrin fragment stabilized NMJs and improved the phenotype of neurotrypsin-overexpressing mice (Hettwer et al., 2014). A recent clinical study identified significantly increased serum levels of the C-terminal agrin fragment in sarcopenic patients as compared to aged matched controls and a national blood donor cohort (Hettwer et al., 2013). Thus, agrin fragments appear to be not only promising candidates in the search for biomarkers in the field of sarcopenia diagnosis, but also carry hope for use as potential therapeutic agents. However, it needs to be mentioned that only about 38% of the sarcopenia patients displayed elevated levels of the agrin fragment in serum, indicating that sarcopenia is indeed likely a multi-factorial disease. This is also supported by the observation that aged transgenic mice lacking neurotrypsin, or overexpressing agrin develop sarcopenia (Bütikofer et al., 2011). Whether agrin signaling is modified or relevant in the context of NMJ maintenance in muscular dystrophies has yet to be explored.

Conversely, when considering second messenger handling as a major determinant of AChR turnover and NMJ continuity, this is definitely aberrant in dystrophic muscle while its role in sarcopenia is much less clear. With respect to dystrophies, alterations in Ca^2+^ and cAMP handling were previously reviewed (Carlson, 1998; Rudolf et al., 2013). Although to our knowledge there are no in-depth reports that have investigated modulation of second messenger signaling upon aging by means of *in vivo* approaches, genomic and proteomic profiling revealed a strong correlation between muscle loss and at least three members of the cAMP signaling system, i.e., adenylate cyclase 2, PKA type Iα, and phosphodiesterase 4a (Ibebunjo et al., 2013). Future work will be necessary to further tighten these links and to understand any possible relationship to age-related alterations of NMJ. Finally, NMJ remodeling and particularly, AChR turnover are affected by the autophagy process. So, what is the contribution of autophagy to sarcopenia and dystrophy? This question has recently been addressed in a nice review (Sandri et al., 2013). In brief, although to date genes that are involved in autophagy have not been found to be directly associated with dystrophinopathies and sarcopenia, autophagy is impaired in muscles of dystrophic mdx mice and Duchenne dystrophy patients (De Palma et al., 2012). Furthermore, treatment of mdx mice with the agonist, AICAR, for the autophagy-driving AMP kinase not only leads to increased autophagic flux but also improves the dystrophic phenotype significantly (Pauly et al., 2012). In general, a fine tuned balance of autophagy seems to be critical for keeping skeletal muscle intact (Neel et al., 2013). While too little autophagic activity might lead to accumulation of damaged organelles and proteins, such as mitochondria (Grumati et al., 2010) or sarcoplasmic reticulum (Russ et al., 2014), exacerbated autophagy would also entail muscle wasting.

## Conclusion

In conclusion, the interplay between neurons and muscle is a principal component that appears to be altered in both sarcopenia and dystrophy. However, the origins of the degeneration in functional interaction between both tissues are likely to be different between those two conditions. Owing to anterograde and retrograde signaling cascades active at the NMJ, it can be envisaged that neuronal degradation may lead to muscle fiber atrophy as well as the reverse of this. Regardless, functional denervation might trigger several pathways leading to the morphological deterioration of NMJs and to altered turnover of AChRs. This could become important in the context of diagnosing and treating sarcopenia, given that with the appearance of agrin fragments, first biomarkers and therapeutic agents for a subset of sarcopenia are also apparent. Since the agrin/MuSK/Lrp4 pathway is a major but not exclusive regulator of NMJ maintenance, further research is needed to better understand these additional physiological signals that decide over NMJ morphology and AChR turnover.

## Conflict of Interest Statement

The authors declare that the research was conducted in the absence of any commercial or financial relationships that could be construed as a potential conflict of interest.
